# Multi-focus image fusion algorithm based on focus detection in spatial and NSCT domain

**DOI:** 10.1371/journal.pone.0204225

**Published:** 2018-09-20

**Authors:** Hongmei Wang

**Affiliations:** School of Astronautics, Northwestern Polytechnical University, Xi’an, Shaanxi, China; Mar Ephraem College of Engineering & Technology, INDIA

## Abstract

Multi-focus image fusion is an effective approach to obtain the all-in-focus image. Focus detection is the key issue of multi-focus image fusion. Aiming at the shortcoming of spatial domain and transform domain algorithms for multi-focus image fusion, a novel multi-focus image fusion algorithm is proposed by combing focus detection in spatial domain and non-subsampled contourlet transform (NSCT) domain. At first, the focused pixels are detected by the sum-modified-Laplacian algorithm in spatial domain. At the same time, the focus detection method is proposed in NSCT domain, namely by MPCNN and voting fusion methods for high-frequency subbands of NSCT. Then, the morphological operation is utilized to correct the focus detection results in spatial domain and NSCT domain. At last, synthesis of detection results is implemented and the fused image can be obtained. Experimental results verified that the proposed algorithm outperformed some state-of-the-art fusion algorithms in terms of both subjective observation and objective evaluations.

## 1 Introduction

Image fusion is one of the most important research subjects in image processing. Nowadays, most research focus on pixel-level image fusion. Pixel-level image fusion is achieved by combing two or more images coming from different or same image sensors so as to get a new image which contains more information than any of the original images and is more favorable for the follow-up working, such as target recognition, image understanding and so on [1].

It is well-known that the camera is limited in focus. An effective way to obtain an all-in-focus image is by image fusion [2]. The key issue in multi-focus image fusion is to detect the focused regions of each source image correctly. Varieties of focus measures have been developed, such as spatial frequency (SF), sum-modified-Laplacian (SML), and Tenenbaum gradient (Tenengrad) [3]. To improve the precision of focus detection results, Zhang [4] presented a novel focus measure method based on graph-based visual saliency algorithm, the watershed and morphological methods.

Basically, there are two types of methods for multi-focus image fusion. One is the spatial domain-based methods, which select pixels or regions from focused parts in the spatial domain to compose the fused images. The above focus measures have been widely applied in spatial domain-based fusion algorithms. Chen [5] proposed a multi-focus image fusion method based on edge model and multi-matting. The edge model and a traditional block-based focus measure are combined to estimate focus maps. In literature [6], orientation information motivated pulse coupled neural network was applied to obtain the initial decision map, and then the mathematical morphology was employed to modify the decision map. SF and the improved alternate SML is applied to build the initial tri-state map in [7] for multi-focus image fusion. Also, inspired by the mechanism of visual attention in humans, saliency detection model is proposed to detect the most noticeable and attractive region in a scene [8]. A novel multi-focus image fusion method based on the regional saliency is proposed in literature [4], in which the focused region of the source image is merged into the fused image as much as possible. Experiments demonstrated that the proposed approach can accurately extract the focused region and is superior to traditional methods in subjective and objective evaluations.

Another type of image fusion methods is the transform domain-based methods [9–11]. Compared with traditional multi-scale transform, MGA (multi-scale geometric analysis) transform can take full advantage of the geometric regularity of image intrinsic structures and obtain the asymptotic optimal representation, so the MGA transform-based image fusion can get better results and attracted more attention. Ridgelet transform, Curvelet transform, Contourlet transform, Shearlet transform [12] and Non-subsampled Contourlet transform (NSCT) [13] have been widely explored in image fusion, especially NSCT [14–17].

The transform domain-based image fusion algorithms are usually composed of three steps: image decomposition, fusion of the low-frequency subband and high-frequency subbands, image reconstruction. The critical element of transform-based image fusion algorithms is the design of fusion rules for subbands. Average or weighted average method is the most commonly used one for the low- frequency subband fusion. For the high-frequency subbands, the most popular fusion rule is to select the coefficients of subbands with larger absolute values. As a result, these rules do not take any consideration of the surrounding pixels. In order to tackle such a problem of traditional rules, some novel fusion rules were proposed for the low-frequency subband and high-frequency subbands [14]. In [17], the modified fusion rule is proposed for low-frequency subband based on SML. Also, a new high-frequency fusion rule based on local Log-Gabor energy is designed. Gao [18] presented a novel multi-focus image fusion algorithm based on non-subsampled Shearlet transform.

Sparse decomposition can represent the salient information of an image by building the relationship between features and sparse coefficients [19, 20]. Most of the sparse representation (SR)-based image fusion methods also belong to the transform domain-based techniques. Unlike the traditional multi-scale transforms that presume the basis functions, SR learns an over-complete dictionary from a set of training images and is proven to be more comprehensive and effective to extract the structure information of the source image. As a result, SR-based algorithm can get fused image with higher quality than traditional multi-scale transform-based algorithms [21, 22]. However, most of the SR-based image fusion algorithms have high computational complexity because of the increased time consumed during the sparse coding.

The above focus measures can be regarded as features. To overcome the shortcoming of artificial feature extraction, deep learning (DL) [23] has been applied in image fusion in recent years. In [24], Liu applied Siamese network to finish image fusion. Experimental results demonstrated that the proposed method can obtain state-of-the-art fusion performance in terms of both visual quality and objective assessment. Deshmukh [25] applied deep believe network (DBN) to obtain the feature vectors of input images. Mean of feature vectors are calculated and multiplied with input source images to obtain the fused image. At the same time, the all convolutional neural network (ACNN) is applied in multi-focus image fusion in literature [26]. Multi-focus image fusion algorithms based on deep learning network involve two key contents: establishment of giant image database and time-consuming training of the network.

No matter the multi-focus image fusion algorithm is implemented in spatial domain or in transform domain, each algorithm has its merit and shortcoming. Spatial domain-based image fusion algorithms have advantageous ability over transform domain-based methods in alleviating blurring effect and eliminating undesirable artifacts [18]. However, the spatial domain-based methods often suffer from block effect and erroneous results at the focused border regions. Although transform domain, especially the high-frequency subbands, can describe the salient features more effectively, transform domain-based fusion algorithms suffer from blurring effect because the results are usually obtained by image reconstruction which modifies the original image information to a certain extent. Inspired by these properties, a novel image fusion scheme by combining spatial information and transformation information is proposed in this paper. The contributions are summarized as follows:

- A focus detection method which combines MPCNN and voting theory is proposed for high-frequency subbands of NSCT.
- The focus detection results in spatial domain and NSCT domain are combined together to get more reliable fused image.
- Modified postprocessing method for focus map is proposed to get high quality fused image.

The remainder of this paper is organized as follows. Brief reviews of NSCT theory and MPCNN are introduced in section 2. In section 3, the proposed algorithm based on focus measure in spatial domain and NSCT domain is described detailed. Experimental results and performance analysis are given in section 4. Finally, the conclusions are presented and future work is detailed in section 5.

## 2 Preliminaries

### 2.1 Non-subsampled contourlet transform

**Fig 1:**
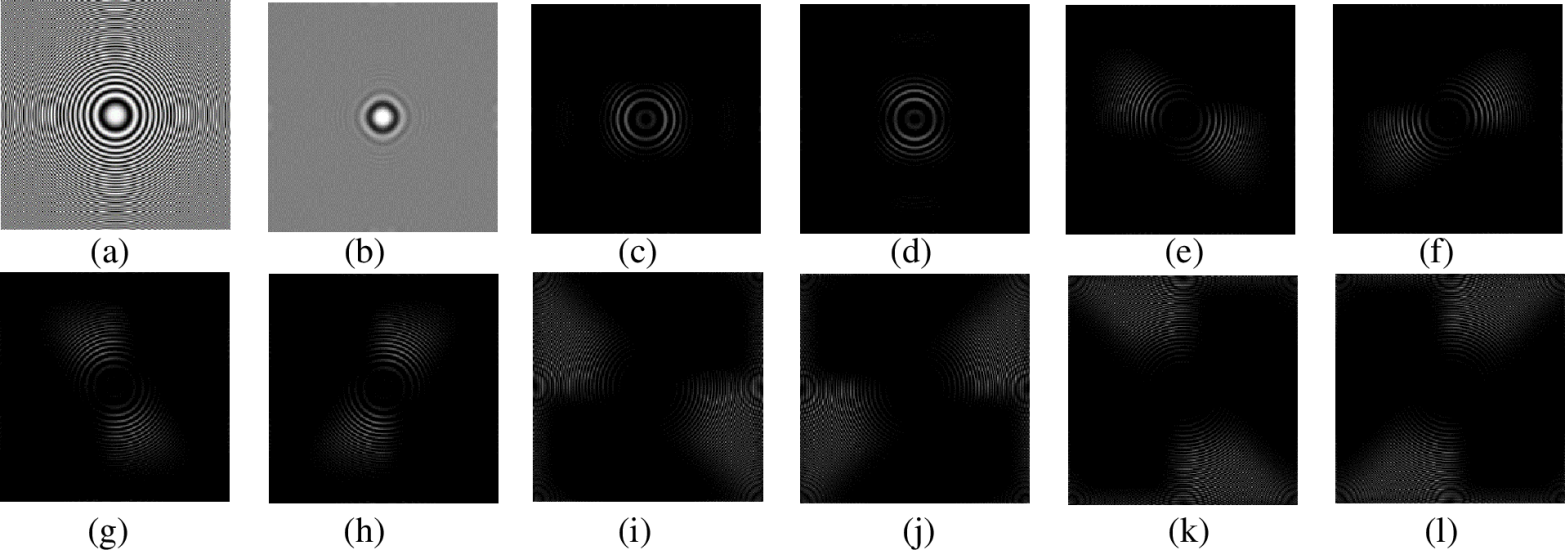
‘zoneplate’ image and its NSCT decomposition results. (a) Original image, (b) Low-frequency subband, (c)-(d) 2-directional high-frequency subbands of the first layer, (e)-(h) 4-directional high-frequency subbands of the second layer, (i)-(l) 4-directional high-frequency subbands of the third layer.

NSCT is a kind of multi-scale and multi-direction computation framework of the discrete images. Compared with other past MGA tools like DWT, NSCT has many important properties [13]:

1. The shift-invariance property thoroughly overcomes the Gibbs effects.
2. The size of subbands is identical, so it is not necessary to require the size of source image is multiples of 2.
3. Identical sizes are very convenient for us to devise the fusion rules for subbands. Refer to reference [13] for the detail theory of NSCT. Fig 1 shows the ‘zoneplate’ image and its NSCT decomposition results. The decomposition level of NSCT is set to 3. The directional number of each level is 2^1^, 2^2^ and 2^2^, respectively.

### 2.2 Memristive pulse coupled neural network

Pulse coupled neural network (PCNN) is a biologically inspired neural network based on the work by Eckhorn [27]. It has been proved that PCNN is very suitable for image processing such as image segmentation, image enhancement, pattern recognition and image fusion [14–16].

Through analyzing the universal phenomenon in biological nerve system and combining with the Eckhorn PCNN, in literature [28], the memristive pulse coupled neural network (MPCNN) is proposed and proved to be more effective than standard PCNN model. The model of MPCNN is given as follows.

$$F_{ij}[n]=I_{ij}+V_{F}\underset{kl}{\sum }M_{ijkl}Y_{kl}[n-1]$$ (1)

$$L_{ij}[n]=V_{L}\underset{kl}{\sum }W_{ijkl}Y_{kl}[n-1]$$ (2)

$$U_{ij}[n]=F_{ij}[n]\left(1+\beta L_{ij}[n]\right)$$ (3)

$$Y_{ij}[n]=\{\begin{array}{cc} 1 & U_{ij}[n]>T_{ij}[n]\\ 0 & U_{ij}[n]\leq T_{ij}[n] \end{array}$$ (4)

$$T_{ij}[n]=M_{ij}(n)+R*Y_{ij}[n]$$ (5)

$$M_{ij}(n)=eM_{ij-1}(n)$$ (6)

Where *I*_*ij*_ is the gray value of the corresponding pixel at position (*i*,*j*) for input image *I*; *L*_*ij*_, *F*_*ij*_ *and U*_*ij*_ are the link input signal, external input and internal behavior, respectively; *T*_*ij*_ and *Y*_*ij*_ are threshold and output of the neuron, respectively. *M*_*ij*_ and *R* are memristor and resistor, respectively. *M*, *W* and *β* are parameters of MPCNN [28].

## 3 The proposed fusion algorithm

The proposed fusion algorithm includes the following four steps.

1. The focus detection in spatial domain is obtained by the SML, and the focus map *Flag*_*S*_ in spatial domain can be obtained.
2. The multi-focus images are decomposed by NSCT, the low-frequency subband and high-frequency subbands are obtained. The focus detection result (*Flag*_*T*_) in NSCT domain is acquired by MPCNN and voting strategy for high- frequency subbands.
3. Synthesizing the focus map *Flag*_*S*_ in spatial domain and the focus map *Flag*_*T*_ in NSCT domain, the final focus map *Flag* can be obtained.
4. The fused image *F* can be obtained by source images and focus map *Flag*.

**Fig 2:**
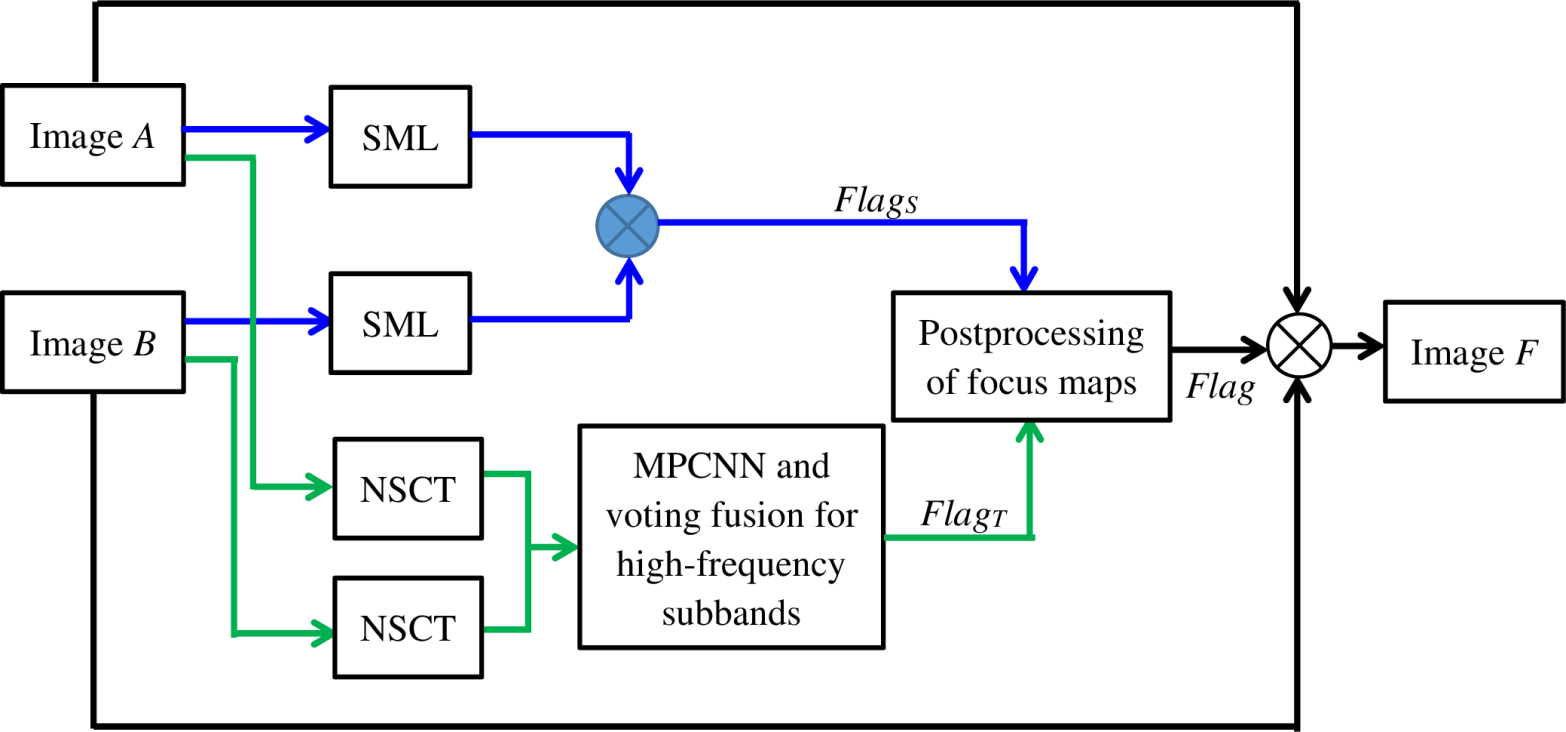
Flowchart of the proposed algorithm.

The detail description is given in section (3.1)-(3.4), respectively. The flowchart of the proposed algorithm is given in Fig 2.

### 3.1 Focus detection in spatial domain

In literature [3], the authors demonstrated that SML can provide better performance than other focus measures, such as Variance, EOG, EOL, SF, and so on. In this paper, SML is applied as a focus measure to detect the focused region in spatial domain.

Suppose *I*_*A*_ and *I*_*B*_ are two multi-focus images to be fused, respectively. The SML of *I*_*A*_ and *I*_*B*_ can be abbreviated as *SML*_*A*_ and *SML*_*B*_, respectively. The focus detection result is obtained by following formula.

$$Flag_{S}(i,j)=\{\begin{array}{ll} 1 & if\;SML_{A}(i,j)>SML_{B}(i,j)\\ 0 & otherwise \end{array}$$ (7)

**Fig 3:**
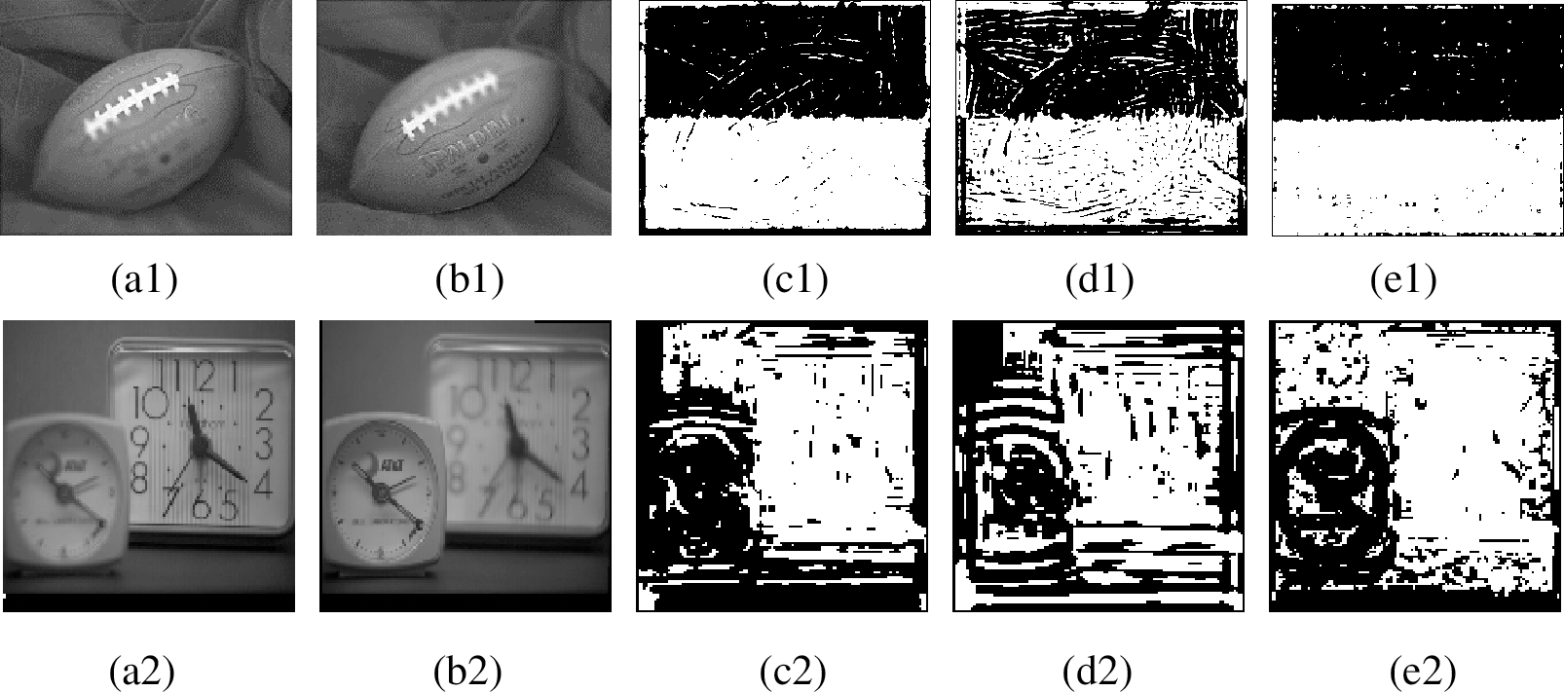
Focus maps by SML for source images and low-frequency subbands. (a1)-(b1), (a2)-(b2) Multi-focus images, (c1)-(c2) Focus maps by SML for low-frequency subbands of NSCT with decomposition level set to 2, (d1)-(d2) Focus maps by SML for low-frequency subbands of NSCT with decomposition level set to 3, (e1)-(e2) Focus maps by SML for source images.

Fig 3 gives focus detection results (focus maps) by SML for low-frequency subbands of NSCT and original source images. Fig 3(A1), 3(B1), 3(A2) and 3(B2) are multi-focus images, respectively. Fig 3(C1) and 3(C2) are focus detection results by SML in NSCT domain, namely for low-frequency subbands of NSCT with decomposition level is 2. Fig 3(D1) and 3(D2) are focus maps by SML for low-frequency subbands of NSCT with decomposition level is 3. Fig 3(E1) and 3(E2) are focus maps by SML in spatial domain, namely using multi-focus source images. From Fig 3, we can find that SML for source images is superior to low-frequency subbands of NSCT.

### 3.2 Focus detection in NSCT domain

Low—frequency subband and high-frequency subbands can be obtained by NSCT transform. From section 3.1, we can find that focus detection result in spatial domain is better than low-frequency subband. High-frequency subbands contain the salient feature of image and are applied to obtain the focus detection result in NSCT domain as a result. Usually, salient features of multi-focus images correspond to lager absolute value of high-frequency subbands coefficients. Therefore, the most popular fusion rule is to select the coefficients with larger absolute values. The shortcoming of this rule is obvious in that it does not take any consideration of the surrounding pixels. In recent years, PCNN is proved to be effective in the fusion of high-frequency subbands and utilized frequently [14]. In this paper, an improved PCNN model, namely MPCNN is utilized as the tool to detect the focused region for high-frequency subbands. Also voting theory is introduced to get more precise detection result.

Supposing $C_{j,k}^{A}(x,y)$ and $C_{j,k}^{B}(x,y)$ are high-frequency subbands coefficients at level *j*, direction *k*, position (*x*,*y*) of source image *A* and *B*, respectively. Let $C_{j}^{I}(x,y)$ (*I* = *A* or *B*) denotes sum of absolute value of $C_{j,k}^{I}(x,y)$ at level *j* and all direction *k* (*k =* 1, 2*… N*, *N* is the total directional number at level *j*), namely,

$$C_{j}^{A}=\overset{N}{\underset{k=1}{\sum }}abs\left(C_{j,k}^{A}\right)$$ (8)

$$C_{j}^{B}=\overset{N}{\underset{k=1}{\sum }}abs\left(C_{j,k}^{B}\right)$$ (9)

The process of focus detection by high-frequency subbands in NSCT domain is given as follows.

**Algorithm**: MPCNN and voting-based focus detection for high-frequency subbands of NSCT

1. Initialize parameters, matrices of MPCNN and set the maximum iterative number.

2. **For** each level of high-frequency of multi-focus images *A* and *B*, **do**

        Set total firing map $Y_{j}^{A}=Y_{j}^{B}=0$; feed $C_{j}^{A}$ and $C_{j}^{B}$ into MPCNN as input *I*_*ij*_;

        **For** each iteration, **do**

                Execute MPCNN formulas (1)–(6), obtain firing output *Y*^*A*^[n] and *Y*^*B*^[n] of the network;

                Add up *Y*^*A*^[n] and *Y*^*B*^[n] with $Y_{j}^{A}$ and $Y_{j}^{B}$, respectively

$$Y_{j}^{A}=Y_{j}^{A}+Y^{A}[n]$$ (10)

$$Y_{j}^{B}=Y_{j}^{B}+Y^{B}[n]$$ (11)

        **End for**

        Compare $Y_{j}^{A}$ and $Y_{j}^{B}$, Calculate the focus detection result for level *j* as follow

$$Flag_{j}=\{\begin{array}{ll} 1 & if\;Y_{j}^{A}>Y_{j}^{B}\\ 0 & otherwise \end{array}$$ (12)

**End for**

3. Apply voting theory to focus detection result acquired from all levels and obtain the final focus detection result in NSCT domain, labeled *Flag*_*T*_.

### 3.3 Postprocessing of focus detection results

Although many focus measures have been utilized into the multi-focus image fusion, such as energy, *RMSE*, *SF* and *SML* utilized in this paper. However, determination by above focus measure is insufficient to identify all the focused pixels. In [18], morphological operation is employed to correct the focus detection results. Experimental results show morphological operation alone is not enough for our focus detection results. In this paper, the morphological operation and inconsistent processing are combined together to correct the focus detection results. The specific steps are given as follows.

*Step* 1: Execute morphological operation on *Flag*_*s*_ and *Flag*_*T*_ as follows, respectively.
  $${Flag}_{X}=bwareaopen({Flag}_{X},Areasize)$$ (13)
  $${Flag}_{X}=1-Flag_{X}$$ (14)
  $${Flag}_{x}=bwarpaopen({Flag}_{x},Areasize)$$ (15)
  $${Flag}_{X}=1-{Flag}_{X}$$ (16)
  Where *Flag*_*X*_ can be *Flag*_*s*_ or *Flag*_*T*_. *Areasize* is the size of region in *Flag*_*X*_ to be removed.

*Step* 2: Inconsistent check for *Flag*_*s*_ and *Flag*_*T*_, the result is labeled *Flag*_*I*_.

$$Flag_{I}(i,j)=\left\{ \begin{array}{ll} 1 & if\;Flag_{S}(i,j)\neq Flag_{T}(i,j)\\ 0 & otherwise \end{array}\right.$$ (17)

*Step* 3: Processing of inconsistent focused regions.

From left-top to right-bottom, for all the pixels satisfying *Flag*_*I*_(*i*,*j*) = 1, choose a region of *Flag*_*s*_ and *Flag*_*T*_ centering with (*i*,*j*), abbreviated as *R*_*s*_ and *R*_*T*_. Carry out the following formula.

$$\left\{ \begin{array}{ll} Flag_{S}(i,j)=1-Flag_{S}(i,j) & if\;std\left(R_{S}\right)\neq 0\\ Flag_{T}(i,j)=1-Flag_{T}(i,j) & otherwise \end{array}\right.$$ (18)

Where *std* is standard deviation.

Though above process, the final focus detection result, labeled *Flag*, can be obtained.

$$Flag=Flag_{S}=Flag_{T}$$ (19)

**Fig 4:**
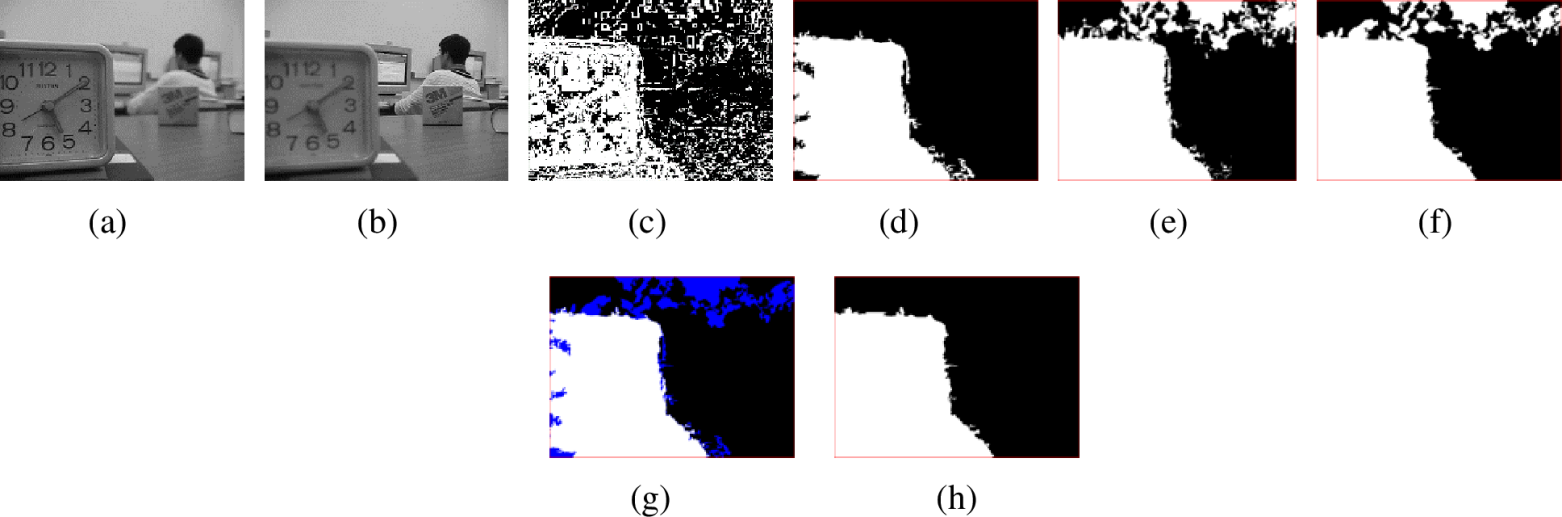
Multi-focus images and focus maps. (a)-(b) Multi-focus source images, (c) Focus map by SML in spatial domain, (d) Morphological operation result of (c), (e) Focus map by MPCNN and voting in NSCT domain, (f) Morphological operation result of (d), (g) Consistent and inconsistent regions for (d) and (f), (h) Final focus map.

Fig 4 shows the total focus detection results for ‘lab’ images. Fig 4(A) is the near focused image and Fig 4(B) is the far focused image. Fig 4(C) is the focus detection result by SML in spatial domain, Fig 4(D) is the morphological operation on Fig 4(C). Fig 4(E) is the focus detection result by MPCNN and voting algorithm in NSCT domain, Fig 4(F) is the morphological operation on Fig 4(E). Fig 4(G) shows the consistent regions (the white and black labeled regions) and inconsistent regions (the blue labeled regions) for Fig 4(D) and Fig 4(F). Fig 4(H) is the final focus map.

From Fig 4(D) and 4(F), we can find decision maps in spatial and NSCT domain are complementary. This is advantageous to obtain accurate focus detection result and higher quality fused image.

### 3.4 Acquirement of the fused image

Supposing *A*, *B and F* are two source images and fused image, respectively. *Flag* is the focus map. The fused image can be obtained by the following formula.

$$F(i,j)=\{\begin{array}{ll} A(i,j) & Flag(i,j)=1\\ B(i,j) & Flag(i,j)=0 \end{array}$$ (20)

The pseudo codes of the proposed algorithm are given as follows.

Input: Multi-focus source images *A* and *B*

Output: Fused image *F*

Begin:

        Compute SML for source images *A* and *B*, and represented as *SML*_*A*_ and *SML*_*B*_, respectively;

        Compare *SML*_*A*_ and *SML*_*B*_, and obtain spatial-domain focus map *Flag*_*S*_;

        Set the parameters of NSCT transform, decompose image *A* and image *B* by NSCT, respectively;

        Compute sum of absolute value $C_{j}^{A}$ and $C_{j}^{B}$ for high frequency subbands;

        Set the parameters of MPCNN;

        Compute and compare outputs of MPCNN for $C_{j}^{A}$ and $C_{j}^{B}$;

        Apply voting theory to obtain the focus map *Flag*_*T*_ in transform domain;

        Execute morphological operation on *Flag*_*S*_ and *Flag*_*T*_, respectively;

        Inconsistent check for *Flag*_*S*_ and *Flag*_*T*_;

        Processing for inconsistent regions and obtain final focus map *Flag*;

        Compute the fused image *F* by *Flag* and source images.

End.

## 4 Experimental results and discussion

In this section, experimental results are given to evaluate the effectiveness of the proposed algorithm. Four databases are applied in ours experiments. (1) The Petrović database [29] which contains 50 pairs of images including aerial images, outdoor images and indoor images. (2) The multi-focus images database [30] which contains 10 pairs of multi-focus images. (3) Lytro multi-focus database [22] which contains 20 pairs of color multi-focus images with size 520×520. (4) The artificial database which is produced by adding Gaussian blur to part of the original images with different standard derivations and Gaussian filter with different size.

**Fig 5:**
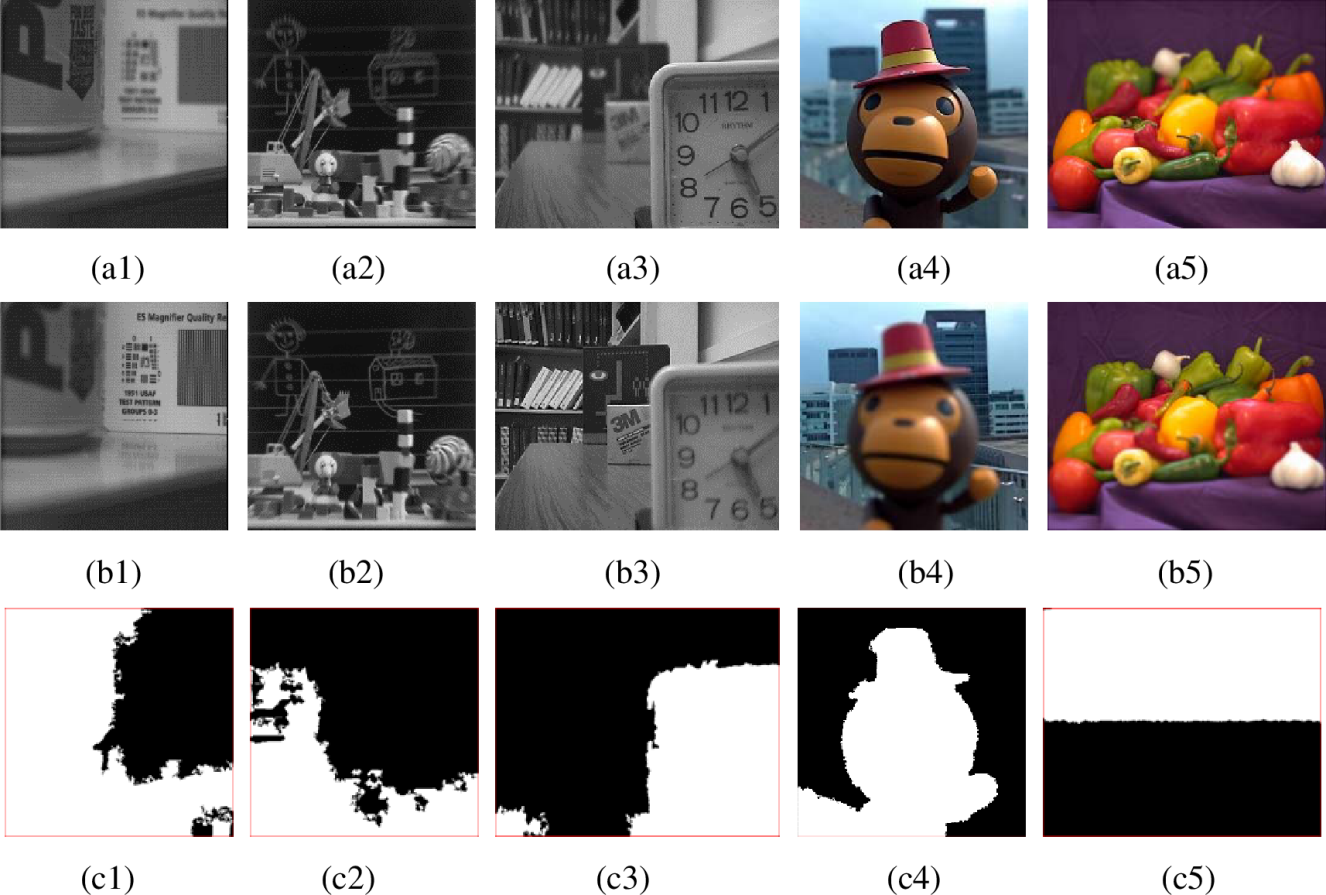
Multi-focus source images and final focus maps. From top to bottom are multi-focus images and focus maps, respectively.

Fig 5 shows some multi-focus images and the corresponding focus maps by ours proposed algorithm. Fig 5(A1) and 5(B1) are ‘pepsi’ images, Fig 5(C1) is the focus map. Fig 5(A2) and 5(B2) are ‘balloon’ images, Fig 5(C2) is the focus map. Fig 5(A3) and 5(B3) are ‘desk’ images, Fig 5(C3) is the focus map. Fig 5(A4) and 5(B4) are ‘lytro-20’ images, Fig 5(C4) is the focus map. Fig 5(A5) and 5(B5) are ‘peppers’ images, Fig 5(C5) is the focus map.

The fusion results obtained from the proposed algorithm are compared with other state-of-the-art image fusion algorithms, including: image fusion algorithm based on spatial frequency-motivated pulse coupled neural networks in nonsubsampled Contourlet transform domain (NSCT-SF-PCNN) [14], multi-scale weighted gradient-based fusion for multi-focus image (MWGF) [10], the guided filter fusion algorithm (GFF) [11], multi-focus image fusion with sparse representation (SR) [19] and the CNN-based multi-focus image fusion method [24]. The codes of the NSCT-SF-PCNN algorithm are available on the author’s homepage [31]. The codes of the MWGF-based method are available on [32]. The codes of the GFF-based method are available on homepage [33]. The codes of the SR-based method and CNN-based fusion method are provided by [34]. In ours algorithm, the parameters of MPCNN are same as literature [28]. The pyramidal and directional filter for NSCT is 'maxflat' and 'dmaxflat7', respectively.

**Fig 6:**
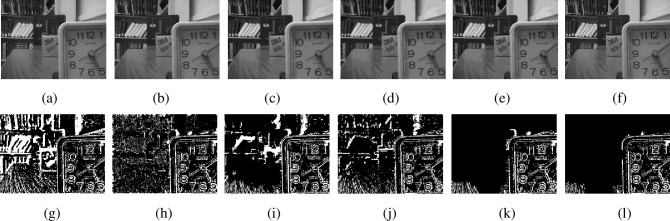
Fused images and residual images by different methods for ‘desk’ images. (a)-(f) Fused images by different methods, (g)-(l) Residual images by (a)-(f) with Fig 5(B3).

Fig 6 presents the experimental results for ‘desk’ images (480×640). Fig 6(A)–6(F) are the results by NSCT-SF-PCNN method, MWGF method, GFF method, SR method, CNN method and ours algorithm, respectively. To show the effectiveness of ours algorithm further, the residual images between the fused images and source image are given in Fig 6(G)–6(L), respectively. The residual image are obtained by subtracting the Fig 5(B3) from each fused image. If the focus regions are selected correctly, the corresponding value of left part pixels of residual image should be zero.

The fusion results of the NSCT-SF-PCNN-based method, MWGF-based method and GFF-based method contain some undesirable artifacts on the fused image, especially for the NSCT-SF-PCNN-based method. This can be demonstrated by the residual images obviously. The SR-based fusion method performs well relatively, but its image quality is still not very high. The CNN method and ours method can select the focused regions most exactly because almost all the pixels in left part of residual images have zero value.

As we know, the fused image can be evaluated by subjective observation and objective evaluation. Objective evaluation also plays important role in image fusion. In this paper, average gradient (*AG*), standard deviation (*STD*), mutual information (*MI*) and edge information preservation value (*Q*^AB/F^) are applied as objective evaluation indexes [35–36]. Usually, the higher value of *AG*, *STD*, *MI* and *Q*^AB/F^, the better quality of the fused image. The objective evaluation results for ‘desk’ images are shown in Table 1.

**Table 1:** Objective assessment of different fusion algorithms for ‘desk’ images.

Metrics	NSCT-SF-PCNN	MWGF	GFF	SR	CNN	Ours
*AG*	5.4073	5.5712	5.4769	5.4311	5.503	**5.5813**
*STD*	46.0508	**46.8873**	46.837	46.5888	46.8171	46.8756
*MI*	6.1778	7.2576	7.0623	7.0154	8.0438	**8.2651**
*Q*^*AB/F*^	0.6598	0.7167	0.7244	0.7024	0.7342	**0.7368**

From Table 1, we can find that the proposed algorithm get the highest value of *AG*, *MI* and *Q*^*AB/F*^. The value of *STD* is a little lower than MWGF-based algorithm. This also demonstrates that the proposed algorithm is the best one compared with other algorithms.

**Fig 7:**
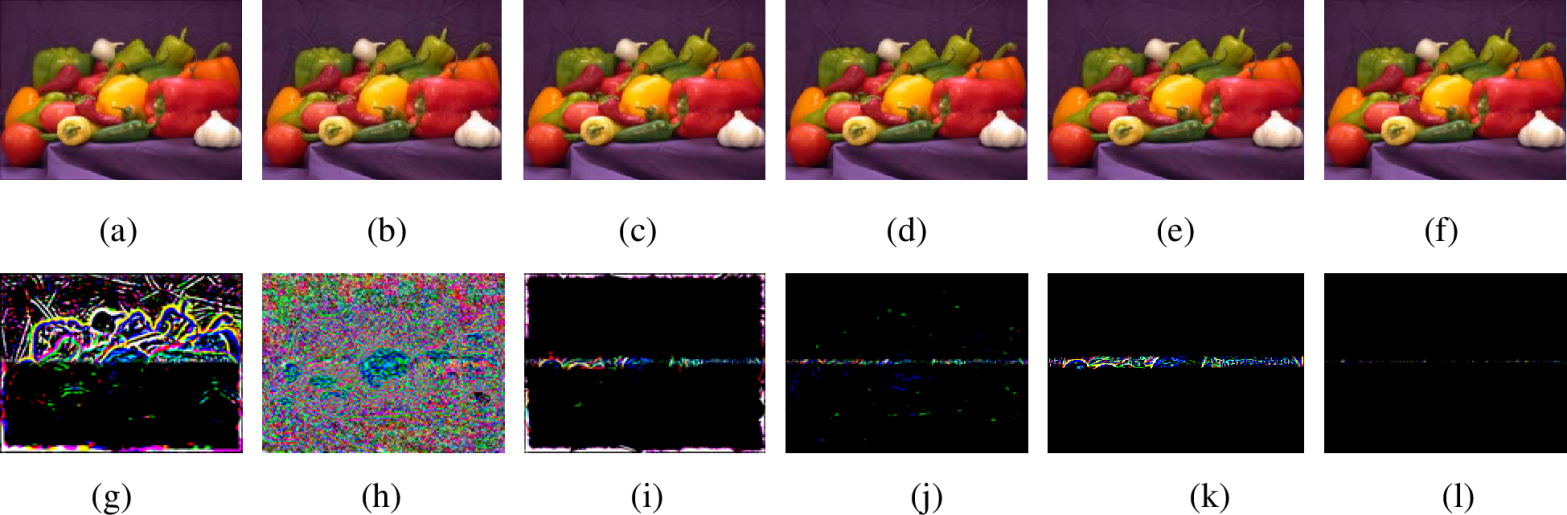
Fused images and residual images by different methods for ‘peppers’ images. (a)-(f) Fused images by different methods, (g)-(l) Residual images by (a)-(f) with all-in-focus image.

Next, image fusion results with all-in-focus image as reference are given to demonstrate the effectiveness of the proposed algorithm further. The all-in-focus image ‘peppers’ (384×512) comes from Matlab toolbox. The multi-focus source images are produced by artificial way and shown in Fig 5(A5) and Fig 5(B5). Fig 7(A)–7(F) are the results by NSCT-SF-PCNN-based method, MWGF-based method, GFF-based method, SR-based method, CNN-based method and ours method, respectively. Fig 7(G)–7(L) are residual images by all-in-focus ‘peppers’ image with Fig 7(A)–7(F), respectively. If the focused regions are selected correctly, the value of pixels of residual images should be zero.

From Fig 7, we can find that most pixels of the residual image by ours algorithm are zeroes. Lots of pixels have non-zero value in Fig 7(G) and Fig 7(H), respectively. Also, some pixels in Fig 7(I), Fig 7(J) and Fig 7(K) have non-zero value. These results also demonstrate that ours algorithm can select the focused region more correctly.

**Fig 8:**
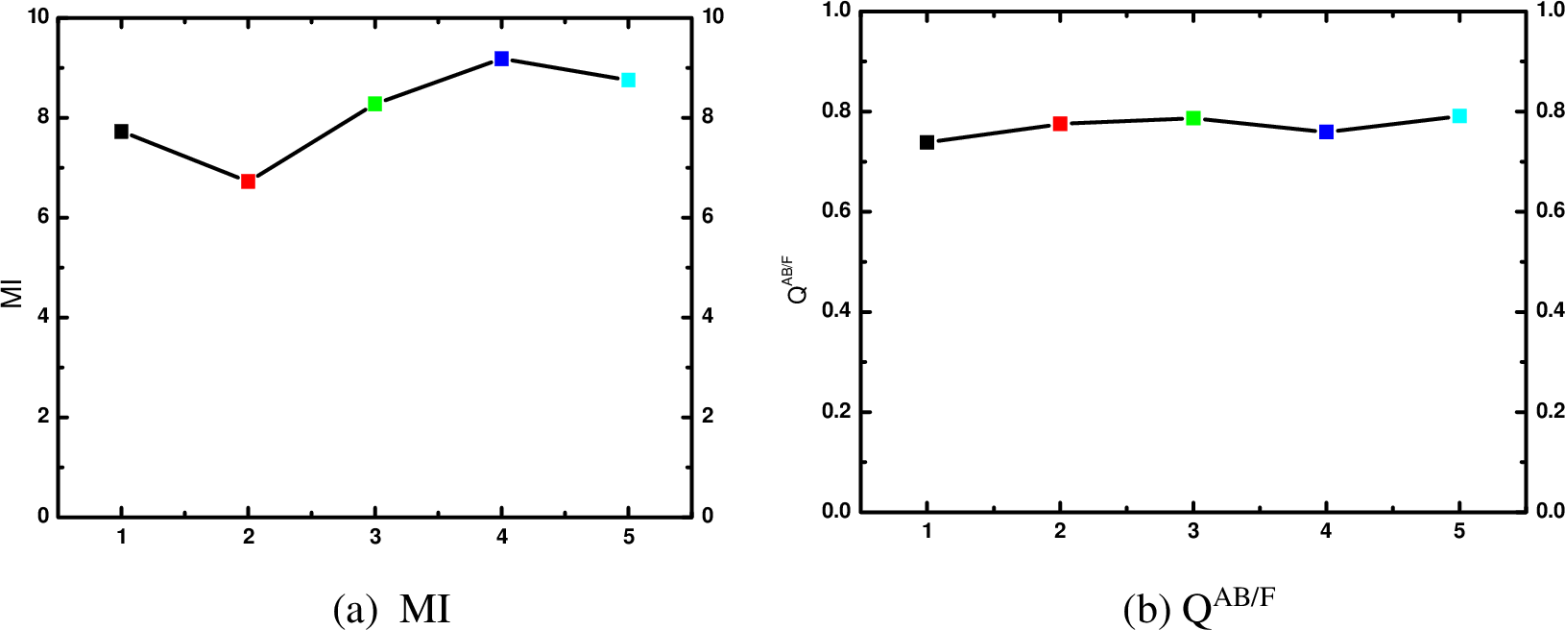
MI and Q^AB/F^ for ‘pepsi’ multi-focus images.

Furthermore, we compare ours algorithm with state-of-the-art literatures for ‘pepsi’ images in Fig 8. Fig 8(A) is the MI of fused image by different algorithms. Fig 8(B) is the Q^AB/F^ of fused image by different algorithms. In this figure, black block represent the result by algorithm of literature [14]; red block is the result by algorithm of literature [15]; green block is the result by algorithm of literature [16]; blue block is the result by algorithm of literature [17] and the last cyan block is the result by ours algorithm.

From Fig 8, we can find that the proposed algorithm can obtain higher MI than the algorithms in literature [14–16], and a litter lower than the algorithm in literature [17]. At the same time, Q^AB/F^ by our algorithm is higher than the algorithms in literature [14–17]. These results also demonstrated that the proposed algorithm is effective for multi-focus image fusion.

At last, the computational performance of the proposed algorithm is evaluated in this section. Table 2 lists the time consuming on the ‘desk’ images of the six algorithms. Because NSCT-SF-PCNN and ours algorithm applied NSCT decomposition which is very time consuming, the efficiency of NSCT-SF-PCNN and ours algorithm are lower. SR-based method takes the longest time because of the sparse coding during the fusion. CNN-based method are also low efficient because of the convolution and pooling operation. In the six algorithms, MWGF-based method is relatively time efficient. GFF-based method costs shortest time and is the most efficient one as a result.

**Table 2:** Time consuming (s) of the six algorithms on ‘desk’ images.

NSCT-SF-PCNN	MWGF	GFF	SR	CNN	Ours
308.642234	8.423734	1.778907	356.706095	302.322311	282.914375

## 5 Conclusions and future work

Pixel-level image fusion has been a very important topic in multi-sensor image fusion. Aiming at obtain an all-in-focus image, a novel multi-focus image fusion algorithm is proposed which combine the property of spatial domain and NSCT domain. In spatial domain, SML is utilized to detect the focused regions. In NSCT domain, MPCNN and voting method are combined together to obtain the focus detection result. At last, by synthesizing the decision maps in spatial domain and NSCT domain, the focus detection result can be obtained and employed to get the fused image. Plentiful experiments are carried through to verify the effectiveness of the proposed algorithm. Experimental results demonstrate that the proposed algorithm outperforms the NSCT-SF-PCNN-based fusion method, MWGF-based fusion algorithm, GFF-based algorithm and some other state-of-the-art image fusion methods in terms of both visual quality and objective evaluation. Although the proposed algorithm can get better fusion result, there is a limit to real-time application. In the future research, we will focus on simplifying the model to improve the efficiency of the algorithm.
